# Bland–Altman agreement analysis between CT predicted and surgical peritoneal cancer index in pseudomyxoma peritonei of appendiceal origin

**DOI:** 10.1038/s41598-023-48975-9

**Published:** 2023-12-06

**Authors:** Mingjian Bai, Jingliang Chen, Yueming Xu, Jing Feng, Ruiqing Ma, Hongmin Jia, Hongbin Xu, Guowei Liang, Hongjiang Wei

**Affiliations:** 1 Department of Clinical Laboratory, Aerospace Center Hospital, Beijing, 100049 People’s Republic of China; 2 Department of Literature and Science, University of Wisconsin-Madison, Madison, WI 50155 USA; 3 Department of Myxoma, Aerospace Center Hospital, Beijing, 100049 China; 4 Department of Radiology, Aerospace Center Hospital, Beijing, 100049 People’s Republic of China

**Keywords:** Gastroenterology, Oncology

## Abstract

Peritoneal cancer index (PCI) is the surgical variable most commonly used to quantify the extent of peritoneal metastases for pseudomyxoma peritonei (PMP) patients. The present study aimed to investigate the agreement between CT predicted and surgical PCI by the *Bland–Altman* method for PMP of appendiceal origin. A total of 167 PMP patients of appendiceal origin were included between 2016 and 2021. *Bland–Altman* analysis was performed for both total PCI and selected PCI (regions 2 + 9–12). After the *Bland–Altman* plot was drawn, the mean bias and its 95% limit of agreements (LoAs) was quantified. Besides, the correlation coefficients between CT-PCI and surgical PCI were also been calculated. The *Bland–Altman* plot showed the mean bias ± SD between total CT-PCI and surgical PCI as 0.431 ± 3.005, with the LoAs from − 5.459 to 6.321. There were nine points of difference in total PCI exceeded the 95% LoAs, with the rate of 5.39% (9/167). As for selected CT-PCI, *Bland–Altman* plot showed the mean bias ± SD between selected CT-PCI and surgical PCI as − 0.287 ± 1.955, with the LoAs from − 4.118 to 3.544. There were ten points of difference in selected PCI exceeded the 95% LoAs, with the rate of 5.99% (10/167). The *Spearman's* rank correlation coefficient between total CT-PCI and surgical PCI was 0.911, *P* < 0.001, as for selected CT-PCI and surgical PCI, the coefficient was 0.909, *P* < 0.001. Although there was a strong correlation for both total and selected CT-PCI with surgical PCI, however, the agreement is still not good in *Bland–Altman* analysis, which suggested that CT-PCI cannot predict surgical PCI accurately even in professional PMP treatment centers. In brief explanation, CT makes it difficult to distinguish the borderline between tumor tissue and mucus and to detect tumor lesions in the small intestine regions, which caused overestimation or underestimation by CT-PCI. In the future, a multiple linear regression model based on CT-PCI might accurately predict surgical PCI preoperatively.

## Introduction

Pseudomyxoma peritonei (PMP) is a rare disease characterized by mucinous ascites and deposits on the peritoneal surface^1^. Cytoreductive surgery (CRS) in combination with hyperthermic intraperitoneal chemotherapy (HIPEC) has been recommended as the optimal treatment for PMP^2^. Peritoneal cancer index (PCI) is the most common used surgical variable which could quantify the extent of peritoneal metastases and determine the feasibility of tumor reduction^3^. Nevertheless, the gold-standard PCI could only be acquired during laparotomy. In clinical practice, computer tomography (CT) remains the reference imaging examination for the evaluation of patients with peritoneal metastases^4^, which might contribute to selecting patients for whom complete resections are achievable and avoid morbid surgery for patients with non-resectable disease. Moreover, CT allows the surgical benefits and risks can be explained to the patient preoperatively^5^.

As far as we know, most studies have assessed the relationship between CT- predicted and surgical PCI by comparing PCI levels or calculating *Spearman's* rank correlation coefficient^6–8^. However, the simple correlation analysis has an important limitation, which does not show whether any difference between two measurements is systematic or random^9^. The *Bland–Altman* analysis was proposed by Martin Bland and Douglas Altman over 30 years ago^10^, which is a popular and widespread means used for analyzing the agreement between two methods, instruments, or raters concerning quantitative outcomes^11^. In this way, *Bland–Altman* plots outperform other methods in that they can reveal both systematic and random errors^9^.

In clinical practice, it is technically difficult to resect massive tumors with wide intra-abdominal involvement, particularly for the selected regions (2 + 9–12) of the small intestine and hepatic hilum^12,13^. Therefore, it is also necessary to assess the agreement between CT predicted and surgical PCI of the selected regions. The present study aimed to investigate the agreement between total CT and surgical PCI in PMP patients of appendiceal origin by the *Bland–Altman* method. Subsequently, we could to decide whether the CT-PCI is acceptable or not in clinical practice. The same procedure was also performed for the selected CT-PCI and surgical PCI.

## Materials and methods

### Patients

This is a retrospective single-center cohort study. The present study was approved by institutional review board (IRB) of Aerospace Center Hospital (20200113-LCYJ-01), and in accordance with the Declaration of Helsinki. Informed consent was obtained from all subjects before surgery. The PMP diagnosis was finally confirmed by two experienced pathologists according to the Peritoneal Surface Oncology Group International (PSOGI) criteria^14^, for patients in 2017 and before, the pathologists reinterpreted the pathological results according to the latest standards.

**Figure 1:**
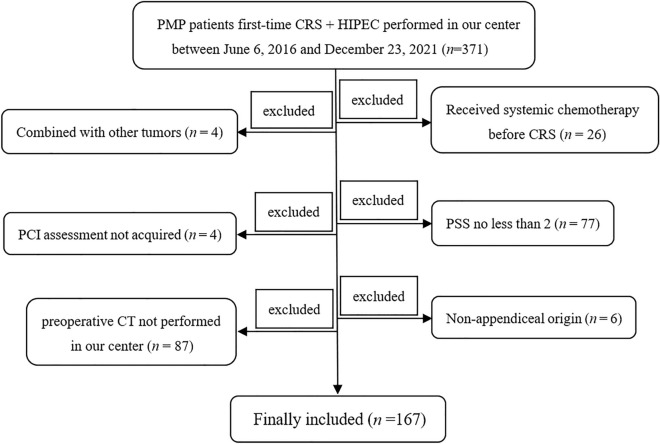
Study enrollment flowchart. A total of 371 PMP patients were retrieved. Patients who combined with other tumors, received systemic chemotherapy before CRS, PCI assessment not be acquired, prior surgical score no less than 2, and of non-appendiceal origin were all excluded. Ultimately, 167 subjects were included. PMP: pseudomyxoma peritonei; CRS: cytoreductive surgery; PCI: peritoneal cancer index; PSS: prior surgical score.

A total of 371 PMP patients whose first-time CRS + HIPEC performed in our center were retrieved from the *EpiData* database between 2016 and 2021, which was built in 2018 and been updated every 3 months. PMP patients whose first-time CRS + HIPEC performed in our center were considered as the inclusion criteria. The exclusion criteria including the following aspects: ① Combined with other tumors (one with nasopharyngeal carcinoma, one with oral cancer, one with breast cancer, and one with both thyroid cancer and breast cancer, total *n* = 4); ② Received systemic chemotherapy before CRS (*n* = 26); ③ PCI assessment be hampered during a debulking procedure (*n* = 4); ④ Prior surgical score (PSS) no less than 2 before the standard CRS + HIPEC (47 with PSS 2, and 30 with PSS 3, total *n* = 77); ⑤ Preoperative CT not performed in our center (*n* = 87); ⑥ Patients of non-appendiceal origin (three originated from colon, two originated from ovary, and one originated from gallbladder, total *n* = 6). Finally, a total of 167 PMP subjects were included in the present study (Fig. 1).

### Enhanced CT procedure

Preoperative CT examination was performed by GE LightSpeed CT (VCT) and SOMATOM Siemens Force dual-source CT equipment. The median time span between CT examination and operation was 7 (5, 9) days. In PMP patients, mucinous tumors often implants into the small intestine region, which always determines whether the surgery can achieve a complete cytoreduction. In order observe the situation of small intestine involvement clearly, meglumine diatrizoate orally and iopromide intravenously were all adopted when patients underwent enhanced CT examination.

Contrast media injection protocol: First, patients orally took fixed 150 ml meglumine diatrizoate (Xi’an Han Feng, China), then, iopromide (Bayer, Germany) with an iodine concentration of 300 mgI/mL (0.6 g Iodine per kg of total body weight), was intravenously injected at a fixed flow rate of 2.5 mL/s (Iodine delivery rate of 0.75 gI/s) and followed by a 50 mL saline chaser administered at the same flow rate.

#### CT protocol

The scanning range was from the top of diaphragm to the plane of pubic symphysis. The scanning parameters have been set as follows: tube voltage 120 kV, tube current 200 ~ 250 mA, matrix 512 × 512, pitch 1.0, layer thickness and spacing are both 5 mm. Image post-processing with 1.25 mm axial thin layer reconstruction, multiplanar reconstruction, and volume reconstruction.

#### CT-PCI calculation

The total and selected CT-PCI calculation method was same to surgical PCI calculation. The CT was then reviewed by one experienced gastrointestinal radiologist before CRS. It should be noted that there was only one radiologist in our center who was responsible for the preoperative CT-PCI calculation for PMP patients, while other radiologists have less experience in calculating CT-PCI.

### Surgical PCI calculation

**Figure 2:**
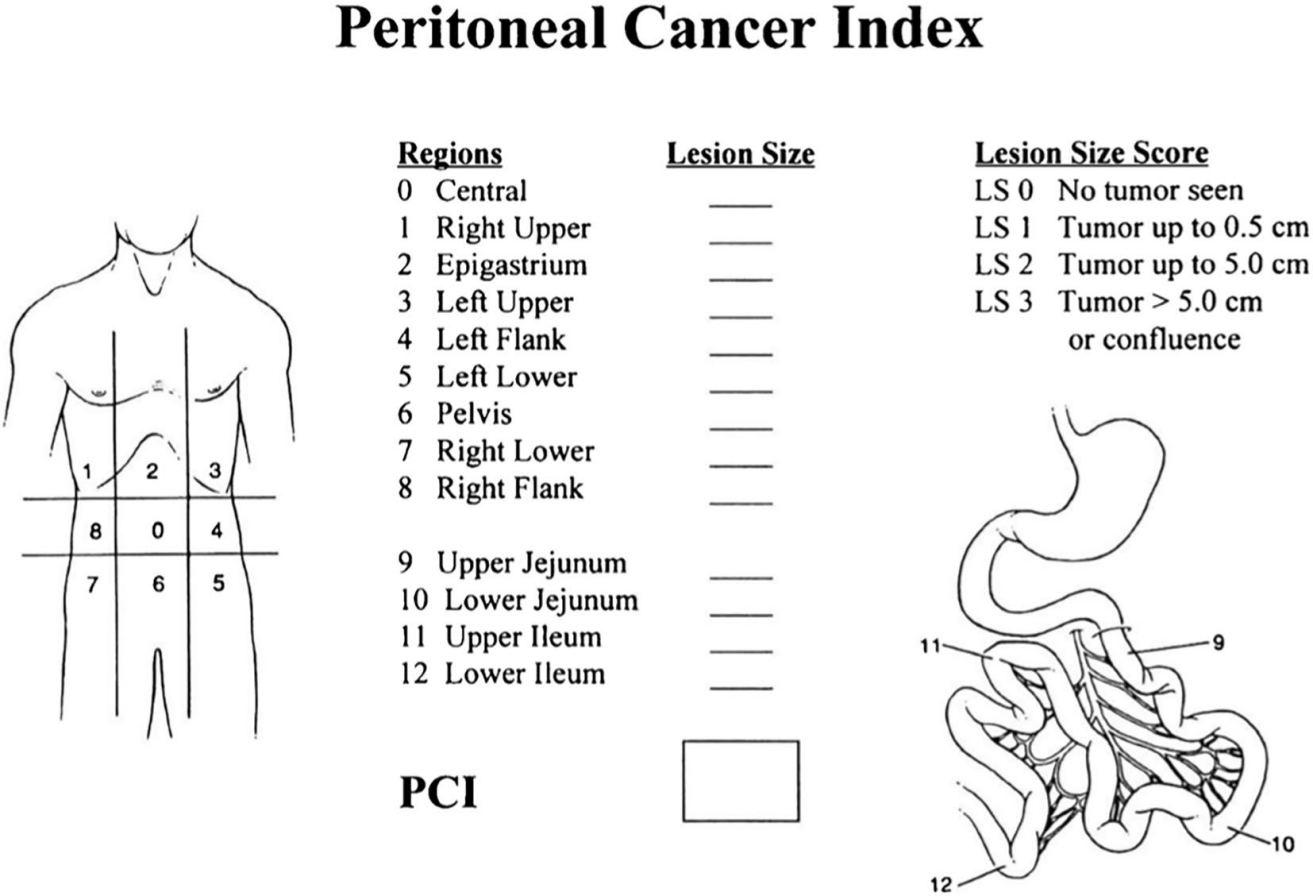
The peritoneal cancer index (PCI) illustration drawn by Harmon and Sugarbaker^27^.

The surgical PCI was served as the gold standard for comparison with CT-PCI. All the operations were performed by four experienced surgeons in our center. The PCI scoring system divides the abdomen into nine anatomical areas with four further areas of the small bowel, refer to Fig. 2^15^. Tumor is assessed in each area and a score of 0–3 is given for each of the 13 areas (0 for no tumor, 1 for nodules < 0.5 cm, 2 for nodules between 0.5 and 5 cm, and 3 for nodules > 5 cm). The total score is then calculated by adding all the scores, with ranges from 0 to 39^16^. Subsequently, the total and selected PCI were calculated respectively. Although the surgeons had known the CT-PCI before the operation, who will remove the visible lesions as much as possible during the operation. In a few cases, some patients were unable to perform surgical PCI score due to huge tumor load.

### Statistical analyses

All statistical analyses were performed using the *SPSS* (version 16.0; IBM Corporation, Armonk, NY, USA) and *MedCalc* (version 15.2.2; MedCalc Software, Flanders, Belgium). All continuous data between groups were compared using the *t* test or *Mann–Whitney U* test, as appropriate.

If both variables x and y are normally distributed, a *Pearson's* correlation coefficient will be calculated for them, otherwise, a *Spearman's* rank correlation will be performed. The correlation coefficient values > 0.7 be regarded as “strong” correlation, values between 0.50 and 0.70 be interpreted as “good” correlation, between 0.3 and 0.5 be treated as “fair” or “moderate” correlation, and any value < 0.30 would be poor correlation^17^.

*Bland–Altman* analysis was performed by the *MedCalc* Software, which quantified the difference between measurements using a graphical method. Requirements for sample size of *Bland–Altman* analysis by the rule-of-thumb of “50 subjects with three replicate measurements on each method”^18,19^ or “at least 100 subjects”^20^. the X-axis represented the average and the Y-axis represented the difference of two measurements. However, use of the LoA assumes that the differences are normally distributed^11^.

Once the *Bland–Altman* graph is drawn, the mean bias and its LoAs was quantified, and the 95% confidence interval (CI) of LoAs was also calculated^21^. In clinical practice, a discrepancy of ± 5–7 PCI points may not be clinically meaningful if it does not influence the ultimate outcome of the surgery^6^, according to this, the acceptable clinical range was − 6 to 6. In this way, if the difference points exceeding LoAs of no more than 5% and the 95% LoAs range falls within the prespecified clinically acceptable range, there is a good agreement between total CT-PCI and surgical PCI, and it can be considered that total PCI can predict the surgical PCI accurately before operation. Additionally, the selected PCI was analyzed separately, however, there was no acceptable clinical range for it.

The present study also aimed to establish the optimal cutoff point of the total and selected CT-PCI to predict surgical resectability for PMP patients. The discriminative ability between total and selected CT-PCI were compared by AUC of ROC curves using a nonparametric approach developed by DeLong et al.^22^. An AUC of 0.9–1.0 indicated an excellent test, 0.8–0.9 indicated a good test, 0.7–0.8 indicated a fair test, 0.6–0.7 indicated a poor test, and 0.5–0.6 indicated a failed test^23^. Two-sided *P*-values less than 0.05 indicated a statistically significant difference.

## Results

The baseline characteristics of the included 167 PMP patients were as follows. There were 79 females and 88 males, and the mean age was 57 ± 12 years. There were 99 patients with PSS 0 while the other 68 patients with PSS 1. The median (*min*, *max*) CT-PCI and surgical PCI were 30 (0, 39) and 28 (0, 39), respectively, paired-sample* t-*test showed *t* = 1.854, *P* = 0.065. The median (*min*, *max*) selected CT-PCI and surgical PCI were 7 (0, 15) and 8 (0, 15), respectively, paired-sample* t-*test showed *t* = − 1.900, *P* = 0.059. There were 62 patients underwent completeness of cytoreduction (CCR) 0/1, while the other 105 subjects underwent CCR 2/3. Details were shown in Table 1.

**Table 1:** Baseline characteristics of the included 167 PMP patients.

Variables	
Sex, *N* (%)	
Female	79 (47.3)
Male	88 (52.7)
Age (years)	57 ± 12
Prior surgical score	
PSS 0	99
PSS 1	68
Total CT-PCI, *Median* (min, max)	30 (0, 39)
Total Surgical PCI, *Median* (min, max)	28 (0, 39)
Selected CT-PCI, *Median* (min, max)	7 (0, 15)
Selected Surgical PCI, *Median* (min, max)	8 (0, 15)
Degree of radical surgery, *N* (%)	
CCR (0/1)	62 (37.1)
CCR (2/3)	105 (62.9)
Histopathologic classification, *N* (%)	
DPAM	137 (82.0)
PMCA	23 (13.8)
PMCA-S	7 (4.2)

PMP: pseudomyxoma peritonei; PSS: prior surgical score; PCI: peritoneal carcinomatosis index; CCR: completeness of cytoreduction; DPAM: disseminated peritoneal adenomucinosis; PMCA: peritoneal mucinous carcinomatosis; PMCA-S: peritoneal mucinous carcinomatosis with signet ring cells.

**Figure 3:**
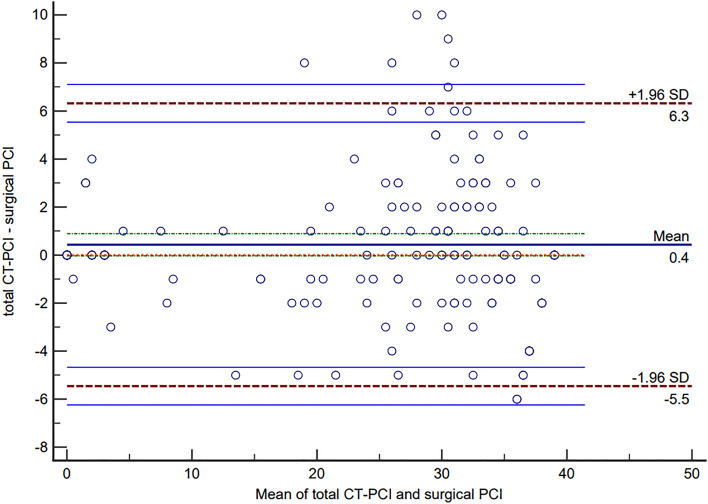
Bland–Altman plot for agreement analysis between total CT-PCI and surgical PCI (*n* = 167). Limits of Agreement are shown as dashed, purplish red lines with 95% confidence intervals (light blue lines), and bias (as solid blue line) with 95% confidence intervals (green dashed lines).

The *Spearman's* rank correlation coefficient between total CT-PCI and surgical PCI was 0.911, *P* < 0.001. Although the differences between total CT-PCI and surgical PCI suggested not accord with normal distribution by *Kolmogorov–Smirnov* methods (*Z* = 2.401, *P* < 0.001), a histogram showed approximate normal distribution (supplement Fig. 1). The *Bland–Altman* plot showed the mean bias ± SD between total CT-PCI and surgical PCI as 0.431 ± 3.005, with LoAs were − 5.459 to 6.321. The 95% *CI* of lower LoA was − 6.245 to − 4.672, and the 95%*CI* of upper LoA was 5.535 to 7.107 (Table 2). There were nine points of difference in total PCI that exceeded 95% LoAs, with the rate of 5.39% (9/167) (Fig. 3).

**Table 2:** Bland–Altman descriptive statistics between total CT-PCI and surgical PCI.

	Value
Sample size	167
Mean (total CT-PCI)	23.719
Mean (total surgical PCI)	23.283
Arithmetic mean (difference)	0.431
95% *CI* (mean difference)	− 0.028 to 0.890
*P* (H0: mean = 0)	0.065
Standard deviation (difference)	3.005
LoAs	− 5.459 to 6.321
95% *CI* (lower limit)	− 6.245 to − 4.672
95% *CI* (upper limit)	5.535 to 7.107

PMP: pseudomyxoma peritonei; PCI: peritoneal carcinomatosis index; LoA: limit of agreement.

**Figure 4:**
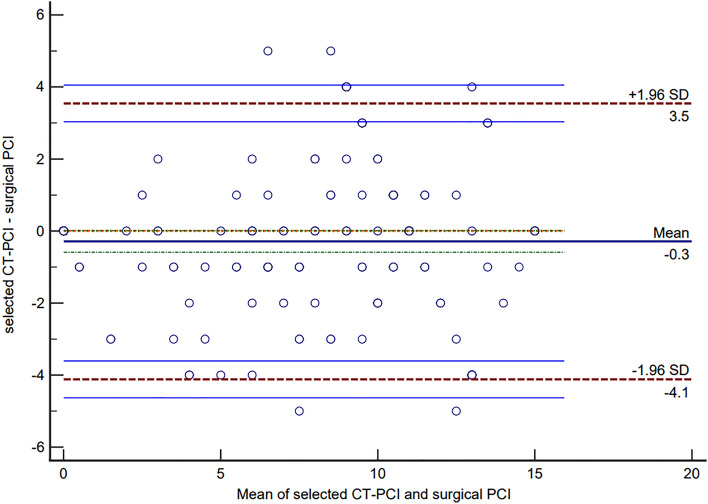
Bland–Altman plot for agreement analysis between selected CT-PCI and surgical PCI (*n* = 167). Limits of Agreement are shown as dashed, purplish red lines with 95% confidence intervals (light blue lines), and bias (as solid blue line) with 95% confidence interval (green dashed lines).

The *Spearman*'s rank correlation coefficient between selected CT-PCI and surgical PCI was 0.909, *P* < 0.001. Although the differences between selected CT-PCI and surgical PCI suggested not accord with normal distribution by *Kolmogorov–Smirnov* methods (*Z* = 2.843, *P* < 0.001), a histogram showed approximate normal distribution (supplement Fig. 2). The Bland–Altman plot showed the mean bias ± SD between selected CT-PCI and surgical PCI as − 0.287 ± 1.955, with LoAs were − 4.118 to 3.544. The 95% *CI* of lower LoA was − 4.630 to − 3.607, and the 95% *CI* of upper LoA was 3.032 to 4.055 (Table 3). There were ten points of difference in selected PCI that exceeded 95% LoAs, with the rate of 5.99% (10/167) (Fig. 4).

**Table 3:** Bland–Altman descriptive statistics between selected CT-PCI and surgical PCI.

	Value
Sample size	167
Mean (selected CT-PCI)	6.808
Mean (selected surgical PCI)	7.096
Arithmetic mean (difference)	− 0.287
95% *CI* (mean difference)	− 0.586 to 0.011
*P* (H0: mean = 0)	0.059
Standard deviation (difference)	1.955
LoAs	− 4.118 to 3.544
95% *CI* (lower limit)	− 4.630 to -3.607
95% *CI* (upper limit)	3.032 to 4.055

PMP: pseudomyxoma peritonei; PCI: peritoneal carcinomatosis index; LoA: limit of agreement.

**Figure 5:**
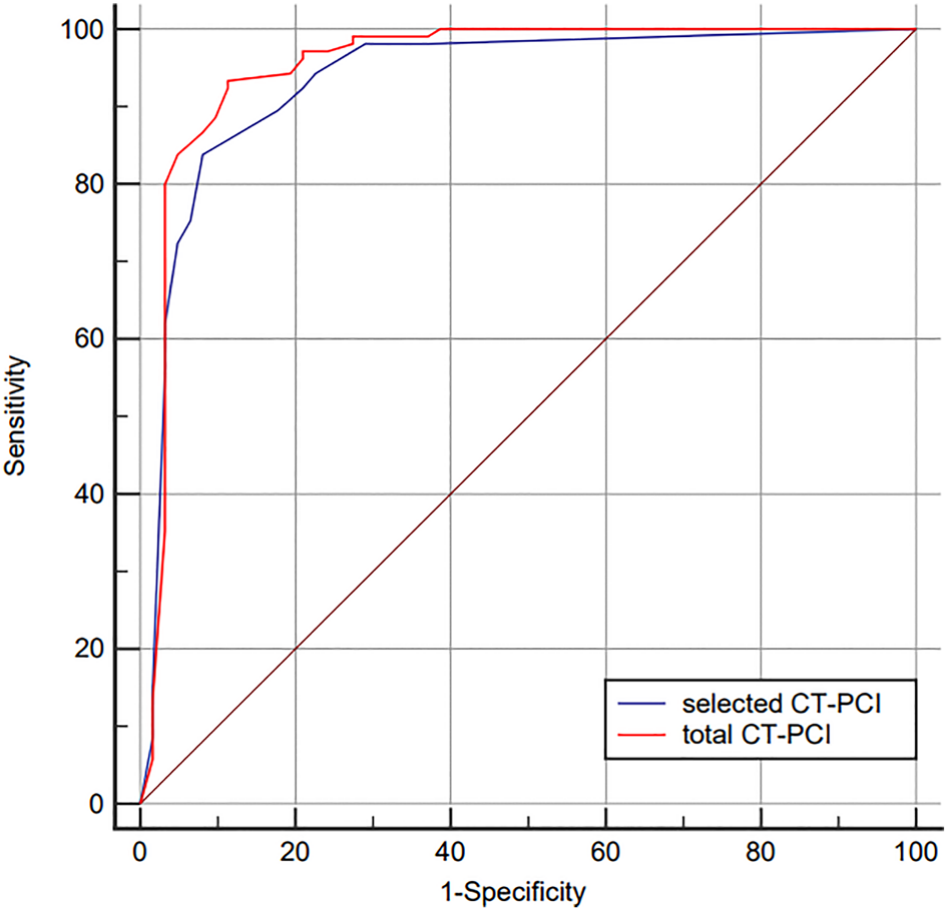
The comparison of ROC-AUC between total CT-PCI (AUC = 0.952) and selected CT-PCI (AUC = 0.934) for predicting surgical resectability in PMP patients (*P* < 0.001).

Taking the "completeness of cytoreduction" as the gold standard, the ROC-AUC of total CT-PCI in predicting surgical resectability was 0.952 (95% *CI* 0.908–0.979), with the sensitivity of 93.33% and specificity of 88.71%. The ROC-AUC of selected CT-PCI to determine surgical resectability was 0.934 (95% CI 0.886–0.967), with the sensitivity of 83.81% and specificity of 91.94%. The discriminative ability of total and selected CT-PCI in predicting surgical resectability was compared by the method of DeLong et al. (*Z* = 2.238, *P* = 0.025). Details are shown in Table 4 and Fig. 5.

**Table 4:** Details of total and selected CT-PCI to predict surgical degree for PMP patients.

	AUC (95 *CI*%)	Cutoff point	Sensitivity (%)	Specificity (%)	*Youden* Index
Total PCI	0.952 (0.908–0.979)	24	93.33	88.71	0.820
Selected PCI (2 + 9–12)	0.934 (0.886–0.967)	6	83.81	91.94	0.758

PCI: peritoneal carcinomatosis index; PMP: pseudomyxoma peritonei.

## Discussion

The present study first performed the *Bland–Altman* agreement analysis between CT-PCI and surgical PCI in PMP patients. We found the difference points outside the 95% LoAs between total CT-PCI and surgical PCI with the rate of 5.39%, and the 95%CI of LoAs exceeded clinically accepted range. As for selected PCI, the difference points exceeded 95%LoAs with the rate of 5.99%. This suggested that the agreement between CT-PCI and surgical PCI is still not good enough, the CT-PCI could not predict surgical PCI accurately even in specialized PMP referral center.

In 2015, Flicek K, et al. found the correlation of CT-PCI with surgical PCI was 0.64 in 42 patients with PMP and peritoneal carcinomatosis^6^. In 2020, the United States HIPEC Collaborative performed a multi-center research, which found a moderate correlation between CT-PCI and surgical PCI for patients with noninvasive appendiceal (*r* = 0.689, *n* = 147) and invasive appendiceal (*r* = 0.554, *n* = 121)^7^. The present research found a strong correlation between total or selected CT-PCI and surgical PCI (all *r* > 0.900), which seems that the correlation is much higher than the previous studies. The most important reason is that the specialized radiologist in our center often participated in the preoperative discussions for PMP patients, who can accumulate rich experience in CT-PCI calculation, nevertheless, there is only one experienced radiologist to undertake this work in our center.

However, correlation quantifies the relationship between numerical variables and may have limitations if used for assessing comparability between methods. It is very likely that two tests designed to measure the same variable be strongly correlated, however, it does not automatically mean that the repeat measurements are also in strong agreement^17^. To our knowledge, the *Bland–Altman* method was first employed to evaluate the consistency between preoperative CT-PCI and surgical PCI in PMP patients, which took into both systematic and random error and is more scientific in evaluating the consistency of quantitative variables. Besides, our research data originated from the largest PMP single center in China^24^, which ensured the sample size of the research. Present study found that there were 5.39% points of difference in total PCI were outside of 95%LoAs, and the 95%*CI* of LoAs exceeded the clinically acceptable range. Although the correlation between total CT-PCI and surgical PCI was strong, the consistency is not ideal, therefore, preoperative CT-PCI could not accurately predict surgical PCI alone. Similarly, the consistency between selected CT-PCI and surgical PCI was not good enough, because there were 5.99% difference points outside 95%LoAs. In all, the CT-PCI could not predict surgical PCI precisely alone before surgery, even for experienced radiologists in PMP referral centers.

Since CT has limited contrast resolution, which always underestimates the extent of disease in patients with peritoneal metastases. The former research confirmed this conclusion in colorectal peritoneal carcinomatosis, which found CT identified the lesion size accurately in 60%, underestimated in 33%, and overestimated in 7% of cases^25^. Interestingly, the present study discovered that the total CT-PCI level appeared to be higher than the surgical PCI in PMP patients, although it did not reach statistical significance. The large amount of mucinous ascites is the typical clinical manifestation of PMP, and the borderline between tumor tissue and mucus cannot be well distinguished during the interpretation of CT results, which may result in slightly higher CT-PCI levels than the surgical PCI. We believe that the Delayed enhanced CT scanning technology may solve the above-mentioned problem to some extent. Oppositely, we found that the selected CT-PCI level appeared to be lower than the surgical PCI, which also did not reach a statistical significant. According to the experience in our center, CT is often difficult to detect tumor lesions in the small intestine regions (9–12) when the lesion is less than 0.5 cm, which may result in an underestimation of PCI. In short, CT manifestations of PMP are different from other peritoneal malignancies when evaluating the tumor burden by CT examination.

In the present study, both total and selected CT-PCI showed an excellent discriminative ability in predicting complete surgical resection for PMP patients. The total CT-PCI was superior to selected CT-PCI, which reached a statistical significant, however, the selected PCI was simpler and time-saving in clinical practice.

There were several limitations in present study. First, there were 87 patients with PMP whose CT examinations were not performed in our center, which might give rise to selection bias in the study to a certain extent. Second, there was only one experienced radiologist responsible for CT-PCI calculation in our center, while in other research centers, the imaging PCI score was often accomplished by two radiologists, so this is another flaw in our research. Third, there were only about one-third of PMP patients underwent CCR 0/1 in our center, we infer that there are many PMP patients in China who do not acquire the right diagnosis in time^26^, which results in excessive tumor load.

## Conclusions

To conclude, although the correlation between the total or selected PCI were excellent for PMP patients, *Bland–Altman* analysis showed the consistency between them were still not good enough. The total or selected PCI alone could not accurately assess the PCI before surgery. The result of our research needs to be further confirmed by other professional PMP referral centers. In the future, a multiple linear regression model including CT-PCI and other predictors might further increase the surgical PCI prediction accuracy.

### Supplementary Information

Supplementary Figure S1.

Supplementary Figure S2.

Supplementary Legends.

## Data Availability

All data generated or used during the study are available from the corresponding author by request.
