# Supplementary figures and images for: Bland–Altman agreement analysis between CT predicted and surgical peritoneal cancer index in pseudomyxoma peritonei of appendiceal origin
Source: Sci Rep. 2023 Dec 6;13:21520. doi: 10.1038/s41598-023-48975-9 (PMC10700599; doi:10.1038/s41598-023-48975-9)

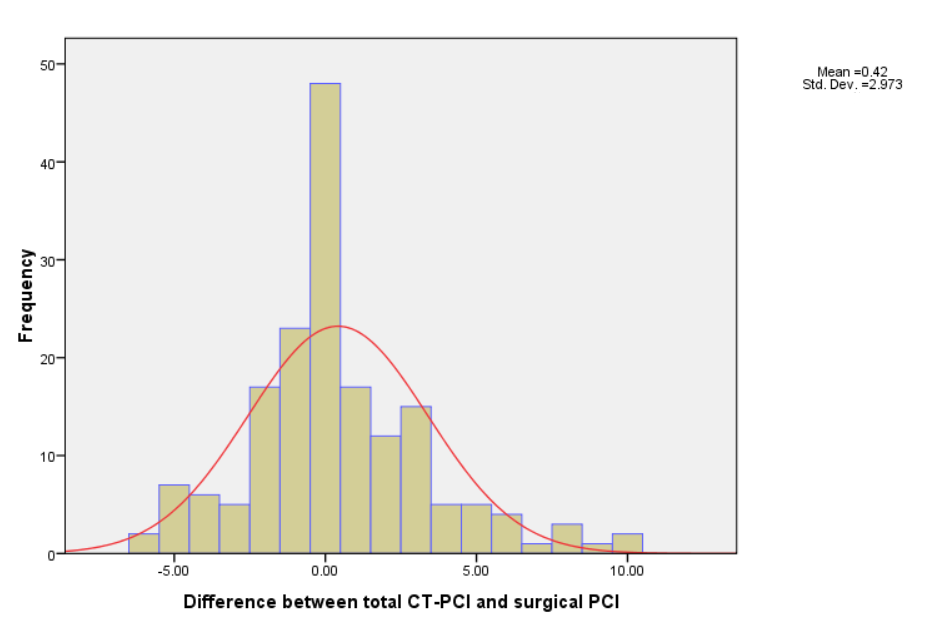

Supplement: Supplementary file 1 — Supplementary Figure S1. [file 41598_2023_48975_MOESM1_ESM.tif]

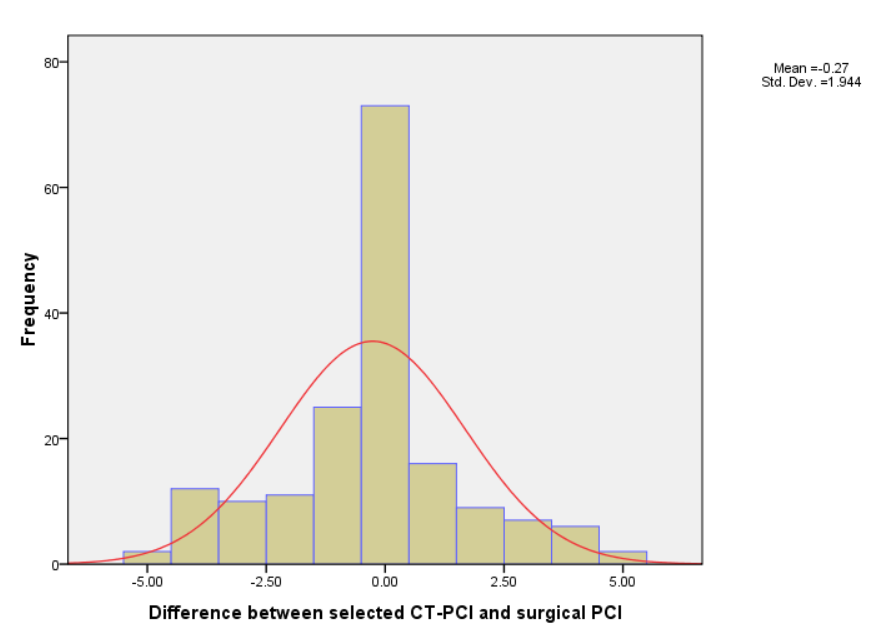

Supplement: Supplementary file 2 — Supplementary Figure S2. [file 41598_2023_48975_MOESM2_ESM.tif]
