# Supplementary material for: Bland–Altman agreement analysis between CT predicted and surgical peritoneal cancer index in pseudomyxoma peritonei of appendiceal origin
Source: Sci Rep. 2023 Dec 6;13:21520. doi: 10.1038/s41598-023-48975-9 (PMC10700599; doi:10.1038/s41598-023-48975-9)
Supplement: Supplementary file 3 — Supplementary Legends. [file 41598_2023_48975_MOESM3_ESM.pdf]

Supplementary Figure 1. The approximate normal distribution of differences points between total CT-PCI and surgical PCI.

Supplementary Figure 2. The approximate normal distribution of differences points between selected CT-PCI and surgical PCI.
